# Management of Fournier's Gangrene in a COVID-19 Patient: Challenges and Dilemmas

**DOI:** 10.7759/cureus.31498

**Published:** 2022-11-14

**Authors:** Diomidis Kozyrakis, Dimitrios Bozios, Anastasios Zarkadas, Georgios Kallinikas, Gerasimos Vlassopoulos

**Affiliations:** 1 Department of Urology, "Konstantopoulio" General Hospital, Nea Ionia, GRC

**Keywords:** covid-19, sepsis, testicular ischemia, necrotizing fascitis, fournier gangrene

## Abstract

Fournier’s gangrene is a urologic emergency manifested as an aggressive form of necrotizing fasciitis. Co-infection of Fournier’s gangrene with COVID-19 might have catastrophic sequelae. We report a case of a 69-year-old male patient, unvaccinated against coronavirus, was obese, and with type 2 diabetes diagnosed with Fournier’s gangrene. Apart from administration of piperacillin/tazobactam and clindamycin, the patient underwent surgical debridement four hours after his presentation. Postoperatively, the PCR test for COVID-19 was proved to be positive. The patients develop septic shock necessitating the delivery of dopamine, supplemental oxygen, and thromboprophylaxis. On the seventh postoperative day, left testicular ischemia was developed and ipsilateral orchiectomy was performed. After his full recovery from an in-hospital infection by *Acinetobacter baumannii*, the patient was transferred to the plastic surgery department. The prompt surgical debridement has likely counterbalanced the health risk originated from COVID-19 infection, contributing to the patient’s full recovery. Testicular ischemia is a very rare condition in necrotizing fasciitis of the genital and perineal space and it could be attributed primarily to the thrombotic nature of coronavirus. Due to the assault of multiple organs and systems, a medical board consisting of urologists and other medical specialties substantially contributed to the favorable outcome.

## Introduction

Fournier’s gangrene is a debilitating, rapidly progressive, and aggressive form of necrotizing fasciitis of the genital and perineal tissues that requires urgent control and treatment. The lesion should be excised in width and depth up to healthy looking tissue with normal blood supply usually in more than one session of surgical debridement, as the extent of necrosis is often broader than what could be initially anticipated [1-3]. Broad-spectrum empirical antibiotic treatment should be administered against all the possible causative bacteria of the gangrene with *Staphylococcus*, *Enterococcus*, *Escherichia coli*, *Pseudomonas*, and anaerobe strains being the most frequent isolates. Blood, tissue, and urinary cultures should be obtained for the etiology to be determined and targeted treatment to be delivered based on sensitivity tests [1,3].

The highly contagious COVID-19 virus, responsible for the coronavirus pandemic, is affecting the global population. As of early October 2022, more than 619 million cases have officially been recorded by WHO and more than 6.5 million deaths were attributed to the complications of the virus [4]. Therefore, due to its vast dissemination, the coexistence of COVID-19 infection with necrotizing fasciitis is deemed to occur with unpredictable and potentially catastrophic results. Herein, our experience with the management of Fournier gangrene in a patient diagnosed with COVID-19 is described and the challenges in diagnostic and therapeutic procedure are highlighted.

## Case presentation

A 69-year-old male patient, unvaccinated against COVID-19 with BMI>35, former smoker, and with type 2 diabetes presented to the emergency department with scrotal swelling and perineal pain, accompanied by fever up 38.5°C for at least 72 hours. 36 hours prior to his initial presentation, he was counseled by his physician to receive fluoroquinolones for orchiepididymitis, but due to the rapid worsening of his condition he was referred to a tertiary center. The body temperature at presentation was 39.2°C, blood pressure was 145/85 mm Hg, and heart rate was 108 beats/min. Physical examination revealed a necrotic lesion of the scrotal skin with a maximum dimension of about 3.5 cm, swelling, tenderness, and crepitus on palpation. He had tachypnea with 27 breaths/min, but the thoracic auscultation was normal. Apart from tachycardia, normal S1 and S2 were revealed after cardiac auscultation. As per protocol, both rapid and formal PCR testing for the detection of COVID-19 were performed. While waiting for the PCR results, the rapid test was negative.

**Figure 1:**
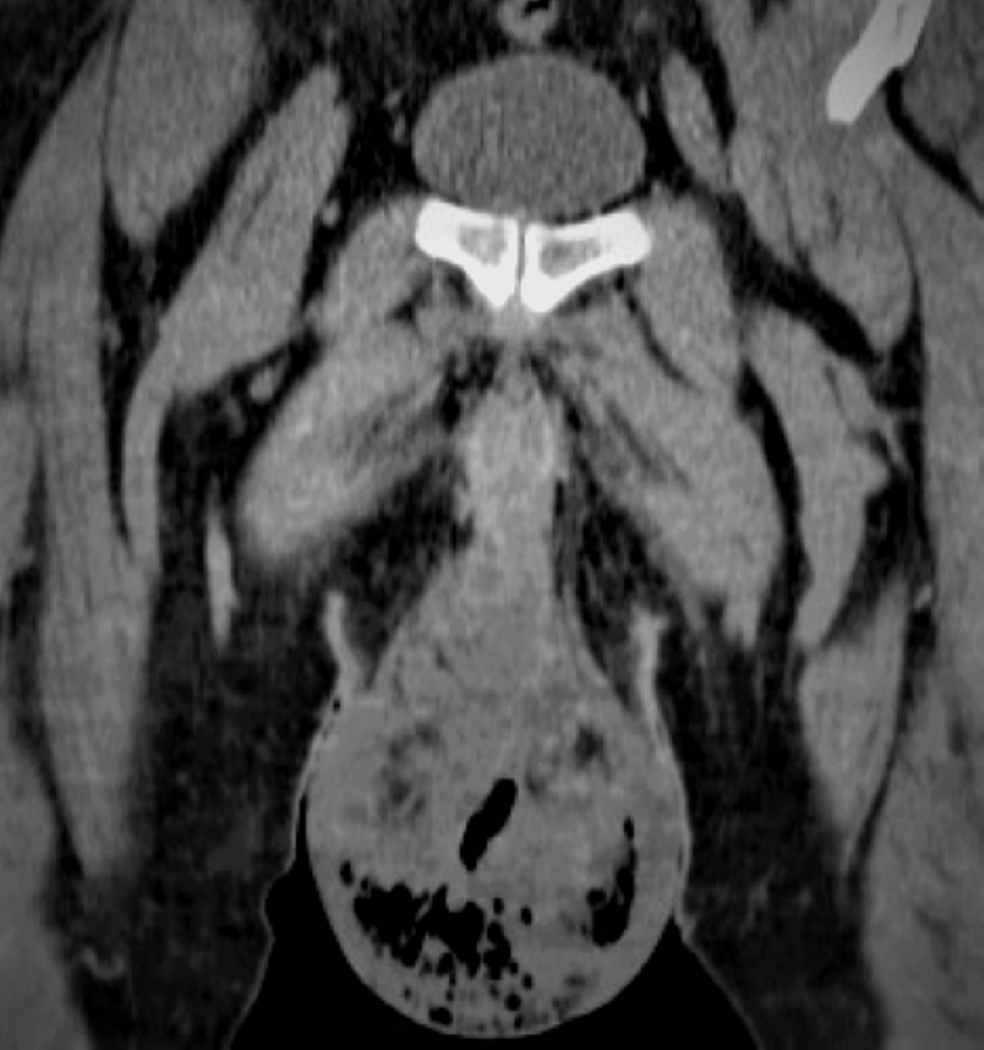
Coronal CT image of the pelvis showing gas- and fluid-containing bilateral scrotal infection.

**Figure 2:**
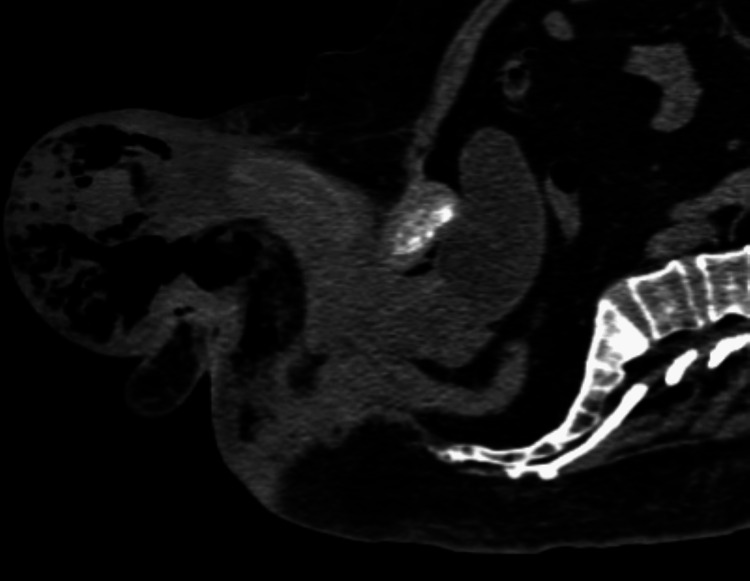
Sagittal image of the Fournier gangrene. Extended gas collection is shown in the scrotum and the superficial layers of the perineum.

Blood tests revealed normal hemoglobin concentration and platelet count but markedly elevated white blood cells (23 × 10^3^/µL, reference range: 4.5-11 × 10^3^/µL), with 86% neutrophilic type. Serum electrolytes and liver biochemistry were normal, while serum creatinine, urea, and glucose level were all elevated (2.4 mg/dL, 94 mg/dL, and 180 mg/dL, respectively). The emergency non-contrast CT scan of the upper and lower abdomen and pelvis was indicative of infection of the base of the penis, the inguinal area, the scrotum, and the perineum (but it was not infiltrating the anal ring) with gas and fluid collection (Figures 1, 2).

Empiric antibiotic treatment was immediately delivered with a combination of piperacillin/tazobactam and clindamycin.

**Figure 3:**
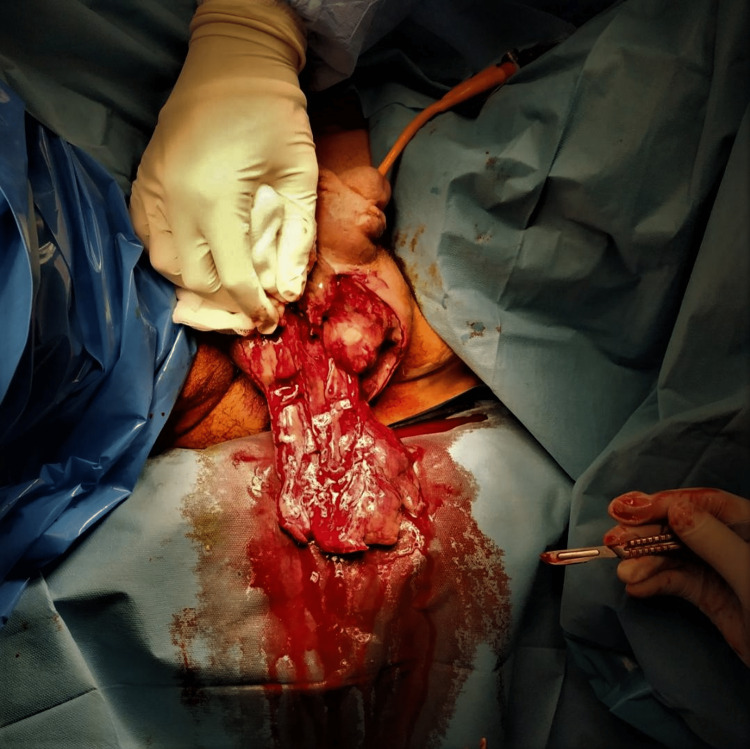
Intraoperative view of the affected area. The layers of both testicles were excised, but the testicles were normal looking and were initially preserved.

Four hours after his presentation, the patient was transferred to the operation theater and, under epidural anesthesia, underwent surgical debridement. Both testes were normal looking and preserved (Figure 3). Postoperatively, the patients develop septic shock with tachycardia, tachypnea, hypotension, metabolic acidosis, and low urine output. Apart from the antibiotic treatment, intravenously delivered dopamine was necessitated for restoration of systolic pressure above 100 mmHg and of urine output greater than 30 cc/h, as well as oxygen treatment with Venturi mask at concentrations of 40% and thromboprophylaxis with low molecular weight heparin (enoxaparin 4,000 U).

During the first postoperative day, the PCR result for COVID-19 became available and was positive for the virus. Afterward, the patient was transferred to the COVID-19 ward supervised by doctors of the urology department, and remdesivir was administered for three days. During his hospitalization, the patient developed atrial fibrillation, persistent hypernatremia, hypokalemia, and hypocalcemia. Surprisingly, no symptoms or signs of pneumonia were detected. Due to the severe distress and his isolation from his family and friends, the patient developed psychosis. For the management of all the aforementioned disorders, the cooperation of urologists with specialists in intensive care medicine, cardiologists, and psychiatrists was fundamental. On the 7th postoperative day and while the improvements of the surgical trauma, mental health, sepsis, and electrolytic disorders were evident, the patient described pain on his left testis, which was also tender and pale. Doppler testicular ultrasonography revealed a testis devoid of blood perfusion. Next day, due to the persistence of ischemia in Doppler imaging, left orchiectomy was performed.

**Figure 4:**
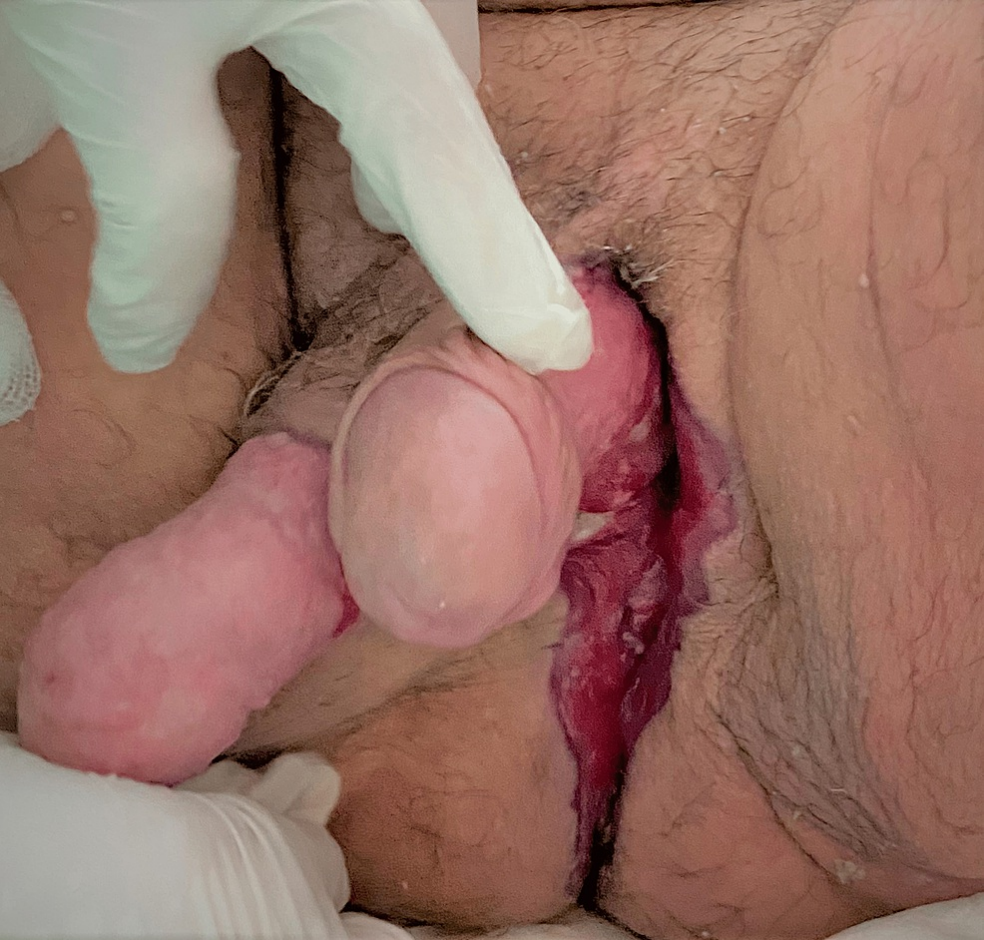
The final surgical outcome after 38 days of hospitalization.

On the 20th postoperative day, a gradual escalation of the body temperature was recorded. Cultures from urine, blood, surgical trauma and venous catheter were collected isolating the same multiresistant *Acinetobacter baumannii* strain in one urine culture and two samples from surgical trauma. Based on sensitivity tests, colistin was administered to the patient for the next 15 days with a rapid recession of the fever and improvement of his condition (Figure 4). Afterward, the patient was transferred to the plastic surgery department for healing of his iatrogenic wound.

## Discussion

It is expected that the coexistence of two severe infections represents a major threat to the patient’s life. It has been reported that any operation performed in a COVID-19 positive patient might increase the risk of morbidity and mortality. As stated by Knisely et al., the perioperative complications were significantly higher in COVID-19 positive versus negative patients (58.3% and 5.6% respectively, p < 0.0001), with cardiac arrest, septic shock, and complications of the respiratory system being the most frequent ones [5]. Our patient developed postoperative sepsis and experienced a prolonged hospitalization but without respiratory complications. Similarly, Silva-Alvarenga et al. reported a case with Fournier gangrene and COVID-19 co-infection in which the respiratory system was also not affected by the virus [6].

It seems that the administration of surgical debridement as early as within 4 hours has counterbalanced the health risk originated from the COVID-19 infection, therefore contributing to the patient’s full recovery. On the other hand, COVID-19 infection in an unvaccinated patient with multiple risk factors (obesity, diabetes mellitus, former smoker) on the grounds of a gangrene is anticipated to have contributed to the dissemination of fasciitis and its progression to severe sepsis [6]. It is our belief that despite the risks of COVID-19 infection, the emergency surgery should not be delayed under the condition that the patients are fully aware of the risks of complications.

Necrosis of soft tissues is attributed primarily to microthrombosis of small arterioles, but testicular ischemia is a rare occurrence in Fournier’s gangrene. Blood supply to the testis comes from a direct branch of the aorta, while the scrotum is perfused primarily by pudendal arterial branches [7]. As Eke stated, only necrotizing fasciitis of the peritoneum or of the retroperitoneal space could justify the ischemia of the testis [7]. In our case, however, the CT imaging did not reveal any abdominal involvement. Therefore, we assume that another etiologic factor could have contributed to the testicular ischemia. The vascular system of the patient, due to obesity, smoking, and diabetes, is expected to have been atherosclerotic long before the onset of the gangrene infection. The prothrombotic and inflammatory effect of COVID-19 is likely a contributing factor that led to the testicular ischemia [8].

The severity of the patient‘s condition necessitated his hospitalization in intensive care unit. However, due to the pandemic, the availability of beds in the unit was limited, and considering that he did not have any need for mechanical ventilation, the patient was treated in the regular COVID-19 ward with enhanced telemetric monitoring. He was under the supervision of the doctors of the urology department, and due to the severity of the disease, consultation from doctors with different medical specialties and level of experience in COVID-19 infection was helpful. In these settings, the close cooperation of urologists with nephrologists, specialists in intensive care doctors, psychiatrists, and cardiologists contributed to a favorable outcome.

## Conclusions

The prompt and thorough surgical debridement of Fournier's gangrene is anticipated to have a favorable impact on the clinical course. Testicular ischemia is a very rare condition in necrotizing fasciitis of the genital and perineal space and could be attributed primarily to the thrombotic nature of COVID-19. Due to the assault on multiple organs and systems, a medical board consisting of urologists and other medical specialties substantially contributed to the favorable outcome.
